# Good data relations key to Indigenous research sovereignty: A case study from Nunatsiavut

**DOI:** 10.1007/s13280-024-02077-6

**Published:** 2024-09-30

**Authors:** Kate M. Ortenzi, Veronica L. Flowers, Carla Pamak, Michelle Saunders, Jörn O. Schmidt, Megan Bailey

**Affiliations:** 1 Mi’kma’ki, the unceded and unsurrendered territory of the Mi’kmaq People, Department of Biology, Dalhousie University, 1355 Oxford Street, PO Box 150000, Halifax, NS B3H 4R2 Canada; 2 The Unceded and Unsurrendered Territory of the Algonquin Anishnaabeg People, 817-315 Holmwood Avenue, Ottawa, ON K1S 2R2 Canada; 3 Nunatsiavut Research Centre, 12 Sandbanks Road, Nain, Nunatsiavut, NL A0P 1L0 Canada; 4 WorldFish, Jalan Batu Maung, Batu Maung, 11900 Bayan Lepas, Penang Malaysia; 5 Mikma’ki, the unceeded and unsurrendered territory of the Mi’kmaq People, Marine Affairs Program, Dalhousie University, 1355 Oxford Street, PO Box 150000, Halifax, NS B3H 4R2 Canada

**Keywords:** Indigenous data sovereignty, Inuit, Nunatsiavut, Research ethics, Science policy

## Abstract

Although researchers are committed to Indigenous data sovereignty in principle, they fall short in returning data and results to communities in which or with whom they conduct their research. This results in a misalignment in benefits of research toward researchers and settler institutions and away from Indigenous communities. To explore this, we conducted a case study analyzing the rate researchers returned data to Nunatsiavut, an autonomous area claimed by Inuit of Labrador, Canada. We assessed the data return rate for all research approved by the Nunatsiavut Government Research Advisory Committee between 2011 and 2021. In two-thirds of projects, researchers did not return the data they had collected. Based on our results and their contextualization with researchers and Nunatsiavut Research Centre staff members, we compiled recommendations for researchers, academia, government bodies, funding bodies, and Indigenous research governance boards. These recommendations aim to facilitate data return, thus putting data sovereignty into practice.

## Introduction

There has been a 20-fold increase in environmental research conducted within Indigenous communities, on Indigenous lands, or in Indigenous waters from 2000 to 2015 (David-Chavez and Gavin 2018 and Jessen et al. 2021). This is in part driven by climate change’s disproportionate effect on Indigenous communities and Indigenous territories (Scott 2008). Concurrently, calls for Indigenous rights within research (termed “research sovereignty”) have increased to include a reevaluation of who decides what research questions are asked, how research is carried out, and who reaps the benefits of research, in the hopes that scientific endeavors better align with international human rights standards (Ignace et al. 2023). The UN Declaration on the Rights of Indigenous Peoples (UNDRIP), Millennium Development Goals, and the Kunming-Montreal Global Biodiversity Framework all emphasize that Indigenous peoples should participate in (not be subjects of) research, control data about themselves and their homelands, and have access to the resultant data to facilitate policy planning, environmental monitoring, and decision making (United Nations 2007; Kukutai and Taylor 2016; Conference of the Parties to the Convention on Biological Diversity 2022). However, this is often not implemented. This adds immediacy to the need for institutionalizing policies and practices that uphold Indigenous data sovereignty.

While these global mandates seem broad in scope, researchers and the institutions to which they belong have obligations to the Indigenous communities in which they work to ensure the principles of data sovereignty translate into the practice of Indigenous data ownership and research sovereignty (Carroll et al. 2020b). By withholding data and results from Indigenous communities, researchers and institutions obstruct Indigenous communities’ rights to make data-driven, informed decisions about how their communities, lands, and waters are managed (ITK 2018), and contribute to ongoing harm and extractive research practices. Therefore, extractive and settler colonialism persist within dominant science as researchers have ultimate control over data and, by extension, with whom and how scientific results are shared and applied (Jennings et al. 2023).

Within what is known by some as Canada, Indigenous (First Nations, Métis, and Inuit) communities are more frequently located in areas experiencing the effects of increased temperatures and are prominently impacted by these changes due to their interrelationship with the environment (NCCIH 2022). Inuit Nunangat, the Inuit homelands in Northern Canada, have experienced warming at a rate about four times faster than the rest of the world over the past four decades (Rantanen et al. 2022). Establishing baseline data to better inform policy decisions and in-turn facilitate resilient Arctic communities is a national and global research priority (Government of Canada, Crown and Indigenous-Northern Affairs 2019; Lee et al. 2023). According to the Canadian government, increased access to environmental data will facilitate greater emergency preparedness, better policy formation within Inuit Nunangat, and make addressing climate change more feasible (Government of Canada, Crown and Indigenous-Northern Affairs 2019). As a result of Arctic research prioritization, research within Inuit Nunangat doubled from 1996 to 2011 (ITK 2018).

The benefits of increased research, however, are not equally received. The increase in research taking place within Indigenous lands and waters benefits researchers by increasing their access to funding and advancing their careers through publications of research dependent on Indigenous data (ITK 2018; Emanuel and Bird 2022; Hudson et al. 2023). Non-Indigenous institutions benefit through increased funding and prestige (Doering et al. 2022). Cumulatively, this has resulted in the accumulation of data from Indigenous communities exported to outside institutions and their benefactors. According to the Tri-Council Policy Statement (TCPS), which establishes policies for the Ethical Conduct for Research Involving Humans, this represents a situation where “a serious imbalance of power prevails between the researcher and participants” that results in injustice (Government of Canada, Interagency Advisory Panel on Research Ethics 2023).

Due to the fraught history of research in Indigenous communities (Smith 1999; Tuck and Yang 2014), there are now several data policy frameworks highlighting the necessity for Indigenous research sovereignty. This term, which encompasses all components of the research cycle and is fundamentally linked to Indigenous rights, includes commitments to data sovereignty, implementing processes to ensure research responds to the needs of Indigenous communities, and incorporating Indigenous collaboration and leadership in research design and execution (Hudson et al. 2023). In this paper, we explore the ideas of data sovereignty and Indigenous data governance, including the ownership of data collected about Indigenous communities, homelands, and waters (CARE Principles 2023). Within Canada, data governance policies include the National Inuit Strategy on Research, the Principles of Ethical Métis Research, the Tri-agency Research Data Management Policy, and the First Nations Principles of OCAP (Ownership, Control, Access, and Possession of data) (NAHO 2010; ITK 2018; Government of Canada 2021; The First Nations Principles of OCAP® 2023). Here, we focus specifically on principles and priorities laid out in the National Inuit Strategy on Research.

### Place-based context

**Fig. 1:**
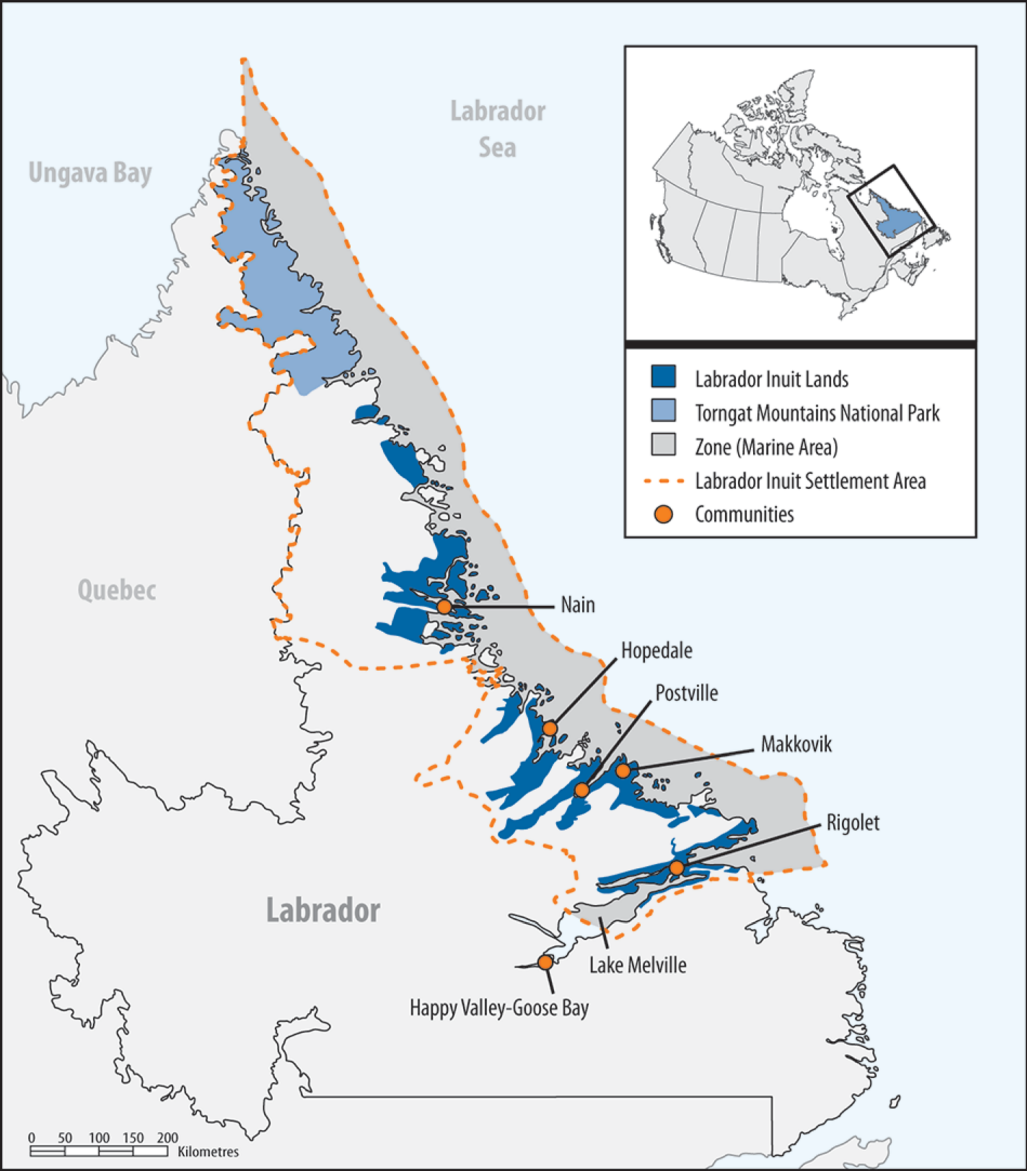
Map of Nunatsiavut and Labrador Inuit Settlement Area Marine Zone. Copy of “Report 3—Implementing the Labrador Inuit Land Claims Agreement” published by the Office of the Auditor General of Canada. This reproduction has not been produced in affiliation with, or with the endorsement of the Office of the Auditor General of Canada

In 2005, the province of Newfoundland and Labrador, Canada, and the Labrador Inuit Association signed the Labrador Inuit Land Claims Agreement (LILCA)—a constitutionally protected treaty stipulating Labrador Inuit sovereignty over Nunatsiavut, or “Our Beautiful Land” in Inuttitut. This resulted in the creation of Nunatsiavut, the Nunatsiavut Government, and the Labrador Inuit Settlement Area (LISA). The LISA includes 72 520 km^2^ land and 48 690 km^2^ adjacent coastal waters (Fig. 1). There are about 7000 Nunatsiavut beneficiaries living throughout Canada with 2600 living in Nunatsiavut (Government of Canada 2018). Nunatsiavut was the first Inuit region in Canada to attain self-government, and is one of four regions that make up Inuit Nunangat, or Inuit homelands in Canada (Path to Self-Government 2023). Inuit are the Indigenous peoples inhabiting Arctic and subarctic Canada, Alaska, Greenland, and parts of Russia (Indigenous People’s Atlas of Canada 2024). Data related to fisheries and wildlife ecology, biology, and harvesting, and environmental data related to physical, biological, and chemical oceanography are all essential to support the Nunatsiavut Government in its self-governance of wildlife policy and domestic harvests (Canada 2010).

### The research application process in Nunatsiavut

**Fig. 2:**
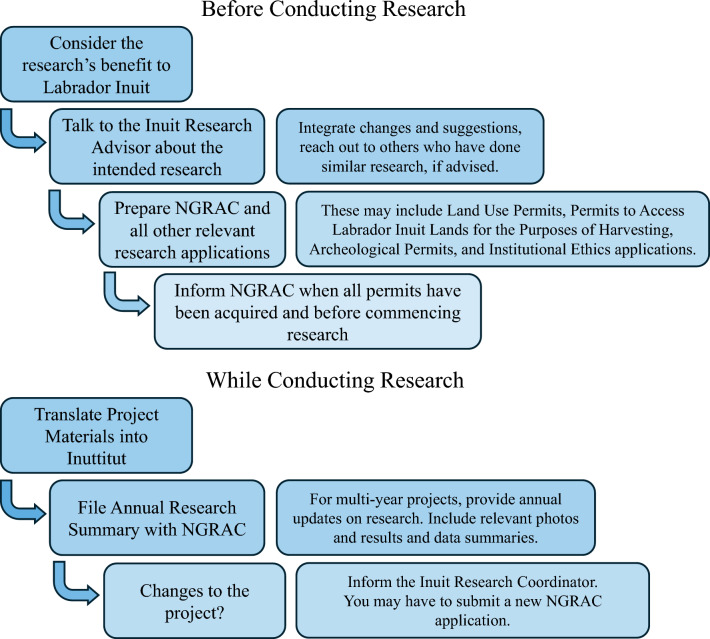
Researchers’ responsibilities in Nunatsiavut. Flowchart of researcher responsibilities before and while conducting research in Nunatsiavut according to the NGRAC application and Inuit Research Advisor. Researcher responsibilities are updated as necessary, and all changes are reflected on the Nunatsiavut Government Research Centre website (Nuesslein et al. 2021; Nunatsiavut 2021)

The Nunatsiavut Government Research Centre was established in Nain, Nunatsiavut, in 2011. At the Centre, Nunatsiavut Government staff work on a variety of research projects led by the Nunatsiavut Government, partner organizations, governmental departments, and visiting researchers (Nunatsiavut 2021). To conduct research within Nunatsiavut, researchers must first contact the Inuit Research Advisor, housed at the Nunatsiavut Government Research Centre, and then complete a research application with the Nunatsiavut Government Research Advisory Committee (NGRAC) (Nunatsiavut 2021) (Fig. 2). The Advisory Committee is made up of representatives from different departments of the Nunatsiavut Government, including the Inuit Research Advisor (ITK 2018). The research applications are reviewed monthly by the NGRAC (Nunatsiavut 2021).

**Fig. 3:**
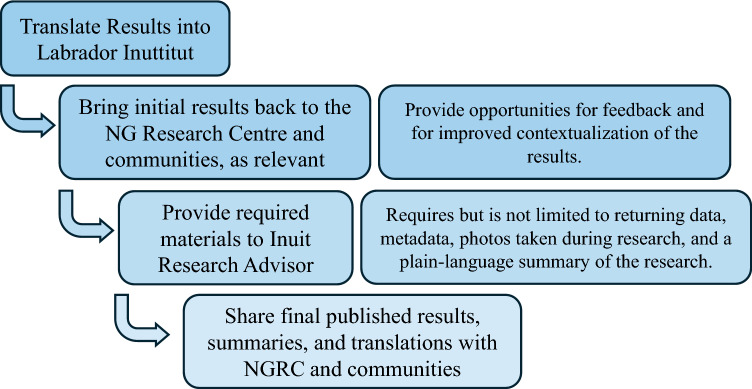
Researchers’ responsibilities after collecting data. Flowchart of researcher responsibilities after collecting data but before publishing results, as outlined in the NGRAC application and research approval letter (Nunatsiavut 2021; Saunders, pers. comm.)

NGRAC research approvals are conditional provided the researchers also: receive research ethics approval from their institutions (human or animal subjects); get all project materials translated into Labrador Inuttitut; and provide to the Government a plain language summary of their research and copies of all research data if the researcher indicates they are able to in their application. Every research project must submit an annual summary of the ongoing research to be included in Nunatsiavut’s Annual Research Compendium. This summary should include a plain-language description of the research, the current status of the research project, and any results to date including photos of the research activities, as appropriate (Nunatsiavut 2021) (Figs. 2 and 3). Although NGRAC approval for research communication is unnecessary, the annual summary should include information about how the research has been shared within Nunatsiavut and to outside audiences. An update to the NGRAC application in 2015 added the explicit question as to whether data would be shared with Nunatsiavut. Research approval is not contingent upon researchers’ ability to share data (Nuesslein et al. 2021).

## Methods

### Case study design

In 2022, the Inuit Research Advisor in Nunatsiavut shared all available approved NGRAC applications and approval letters from 2011 to 2022 with the graduate student researchers. This project did not go through institutional research ethics board review: It was a program evaluation and qualitative improvement study, and therefore did not qualify under TCPS research involving humans (Government of Canada, Interagency Advisory Panel on Research Ethics 2023). It did not go through the NGRAC approval process as it was deemed unnecessary by the Inuit Research Advisor due to its nature as program evaluation and no collection of primary data from Inuit lands, waters, or peoples occurred. We created a simple data frame in Excel cataloging all research projects that had received NGRAC approval from 2011 until 2022 so it could be used and updated by the Inuit Research Advisor after the completion of this case study. We also created a temporary data storage system for research projects that had returned their data. We read each NGRAC application and approval letter, noting the research team’s contact information, research topic, willingness to share data, and NGRAC approval date.

After building the data frame, we contacted representatives of every project that had indicated they would return their data to Nunatsiavut in their initial NGRAC application, but for which we could find no record of data return at the time this study was conducted. Researchers were asked to return their data to the Nunatsiavut Government Research Centre. For email accounts that were no longer active, we attempted to find current email addresses, and forwarded our request to other researchers listed on the NGRAC, if provided. We followed up on our initial request two weeks later for those who had not responded. We included information in the database on investigators’ responses to the request to return data to Nunatsiavut and any follow-up, and we collected all returned data on external hard drives housed at the Nunatsiavut Government Research Centre. We omitted all research projects from 2022 from outreach and analysis on trends in data, as their research was most likely not yet completed, and therefore researchers would not have returned data for these projects.

After creating the data frame of all research projects that had been granted approval, we conducted a literature search to identify other potential research that did not go through the NGRAC approval process during this time period. To do this, we searched Web of Science for published peer-reviewed journal articles with the keywords “Nunatsiavut” or “Northern Labrador” published between 2014 and 2022. We compared author names with the investigators listed on NGRAC applications between 2011 and 2021. For published journal articles that did not have authors in common, we compared article abstracts and keywords to the information provided in the NGRAC applications to find potential matches in case principal researchers had changed over the course of the research project. We report the results of this literature review below; however, we report them separately from the data analysis we conducted on the rate of data returned from researchers that had undergone the NGRAC process.

All the data we collected for this case study were returned to the Nunatsiavut Government Inuit Research Advisor, including all the analysis included herein. This data is owned and maintained by the Nunatsiavut Government Research Centre.

### Research positionality

This research was co-developed between the Nunatsiavut Government Research Centre and two graduate students attending settler academic institutions—a white settler from the USA and a Nunatsiavummiuk from Hopedale, Nunatsiavut. The authorship team therefore is made up of the two students (KMO and VLF), two Nunatsiavut Government Research Centre staff (CP and MS), and two academic supervisors (MB and JOS). KMO and MB are both settler academics working in relation to the lands, waters, and Peoples of Nunatsiavut. VLF, CP, and MS are Labrador Inuit researchers and practitioners, drawing upon their expertise in research and their own lived experiences.

## Results

Between 2011 and 2021, 369 research projects received NGRAC approval representing a wide range of research topics, of which 68 percent were in the natural sciences (Table 1). Of all the initial project proposals, 203 (55%) indicated they would share their data with the Nunatsiavut Government.

**Table 1:** Topics of Approved Research Conducted in Nunatsiavut from 2011 to 2022. Total is greater than 369, the total number of research projects and percent is greater than 100, as several interdisciplinary research projects had more than one topic. Because of inter- and transdisciplinary nature of many projects, it is not feasible to measure the data return rate by research topic. Bolded research topics indicate those in the natural sciences, which account for 68% of research approved in this time period

Research topics	Count	Percent (%)
Health and wellbeing	53	14.2
**Wildlife management and monitoring**	51	13.7
**Marine biology, ecology and conservation**	35	9.4
**Fisheries**	32	8.6
**Climate change**	29	7.8
**Archaeology**	26	7.0
Education and youth	23	6.2
**Contamination and pollution**	21	5.6
Inuit Culture	21	5.6
History	21	5.6
Inuit governance	16	4.3
**Oceanography**	15	4.0
Food security	15	4.0
**Resource extraction**	14	3.8
Language	11	3.0
Women and gender based violence	10	2.7
**Botany**	9	2.4
**Energy**	9	2.4
Tourism	7	1.9
**Geology**	7	1.9
Justice and law enforcement	5	1.3
**Entomology**	2	0.5
Employment	2	0.5
Music	2	0.5
**Glaciology**	2	0.5
Technology and communications	2	0.5
**Invasive ecology**	2	0.5
Art	1	0.3
Genealogy	1	0.3
Housing	1	0.3
Total	445	

**Fig. 4:**
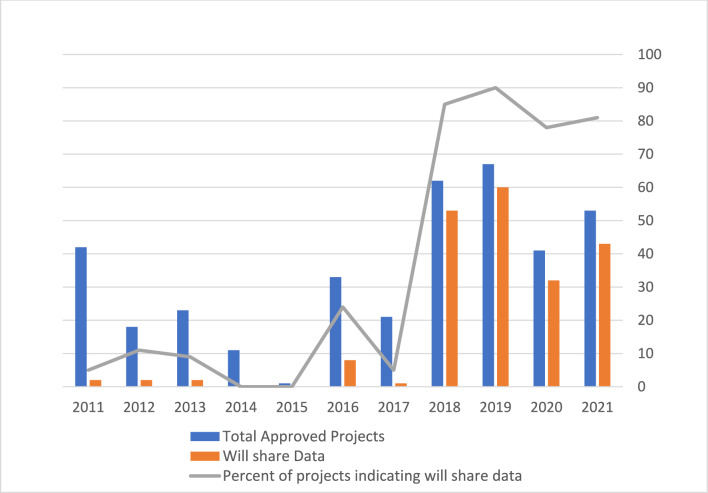
Data Return by Year. NGRAC-approved research projects and percent of those that indicated they would return the data they collected to the Nunatsiavut Government by year

We found that of the 369 applications, 66 research projects had returned their data. This represents one-third (33%) of projects who initially indicated they would return data, and just less than one-fifth (18%) of all approved research projects (Table 2). When projects indicated in their NGRAC applications that they could not return the data, researchers cited small sample sizes and types of investigations that would make de-identifying data impossible or contained protected personal information. However, many projects did not indicate why they could not return their data to the Nunatsiavut Government. Over the decade analyzed, an increasing percentage of projects indicated their intent to return data, likely due to NGRAC adding a question to the research applications asking researchers to indicate if they would return their data to Nunatsiavut in 2015 and the National Inuit Strategy on Research being published in 2018 (Fig. 4).

**Table 2:** Results Summary. Total number of projects that indicated they would return their data compared to those that did return their data

	Number of projects	Percent of total projects (%)	Percent of projects that indicated would return data (%) (203)
Total Projects	369	N/A	N/A
Projects that indicated they would return data in their NGRAC application	203	55	N/A
Total projects that returned data	66	18	33

In correspondence with researchers, 29 indicated that they had not finished data collection. Of the 175 projects that indicated they would share data and had also completed data collection, 30% returned some form of their data or results (53 projects). Most projects did not indicate if they had finished data collection. Data were also supplied by 13 projects that were not finished collecting data. These projects indicated they had returned preliminary data on an annual basis, often consisting of research summaries indicating what data they had collected and where. The majority of projects that submitted these annual research summaries including data told us they had signed research agreements with the Nunatsiavut Government. Three additional research projects had not initially indicated they would be able to share their data in their NGRAC proposal, but then did. These three projects began before the question on returning data was added to the NGRAC application process.

### Trends in returned data

**Fig. 5:**
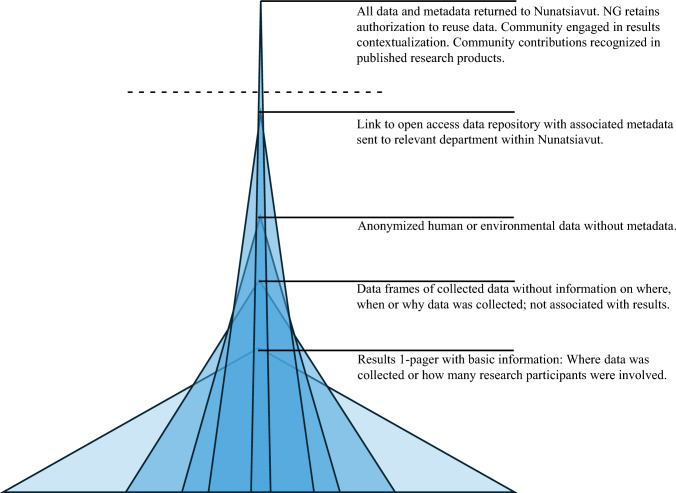
Spectrum of Returned Data. Representation of the types of data that were returned to Nunatsiavut in ascending order from the most frequent types of data returned to the least frequent. Above the dotted line, projects met Nunatsiavut Government Research Centre’s data-related research recommendations and requirements

We took the broadest definition of returning data as possible. If researchers returned any data or results to our request or had returned data or results before our request, we counted the project as having ‘returned data’ for the purposes of our analysis, as long as the results included a link to an accessible data repository or supplemental materials that outlined the data and metadata captured. The majority of returned data were low quality and demonstrated limited usefulness due to a lack of metadata. Among the variation of returned data, most consisted of a one-page research summary only. In other cases, researchers sent published peer-reviewed articles that provided open-source data access information. When whole data frames or spreadsheets were submitted, they were often not accompanied by metadata or descriptions of data collection processes, which greatly limited the data’s usefulness. In several cases, researchers provided links to other data portals where the data was housed, such as within Canadian government departments, academic institutions, and non-profit organizations. Although this is not returning data directly to Nunatsiavut, we included these projects because the data was accessible to the Nunatsiavut Government, if only indirectly (Fig. 5). In email correspondence and conversations with past research investigators, we noted a generalized hesitancy to return the data to Nunatsiavut Government among researchers who had previously indicated their intent to do so. Several researchers asked for follow-up conversations to explain the Nunatsiavut Government’s intention in collecting their data. These trends are consistent with other research documenting limited care of Indigenous data due to a lack of metadata, mislabeled metadata, no record of permissions given by Indigenous rights holders for use of their knowledge, and protocols for data usage (Carroll et al. 2020b; Hudson et al. 2023; Jennings et al. 2023).

### Trends in unreturned data

In cases of unreturned data, lower-than-expected sample sizes or the type or specificity of collected information made it impossible to de-identify research participants; therefore, researchers indicated they did not feel it would be ethical to return data. In many cases, too much time had passed, and the data were destroyed in accordance with pre-stated data management policies. Other reasons data could not be returned included a lack of knowledge as to where the data in question were located, or if they still existed, a lack of internal project or research group processes indicating who had authority to return the data, and an inability for this research team to get in contact with the primary investigators from the initial research project. Some researchers never completed their projects, and therefore said they could not supply the data they had collected. Most frequently, researchers responded that they would only be able to return the data after publication.

In our literature search, we identified 10 published research articles from between 2014 and 2022 in which there was no identifiable connection to any approved NGRAC, despite the work falling under the purview of the Nunatsiavut Government. Of these peer-reviewed journal articles, nine were in natural sciences.

When researchers receive NGRAC approval, they are sent an approval letter outlining their continuing responsibilities. These responsibilities include getting results translated into Labrador Inuttitut, providing a plain language summary of their research, and providing copies of all published materials to the Inuit Research Advisor upon the project’s completion (Fig. 3). We did not track the researchers’ adherence to meeting these obligations. However, the Nunatsiavut Inuit Research Advisor informed us that in their estimation, about five percent of researchers send them copies of all published materials that resulted from the research and about 20 percent provide a plain language summary of their research. They indicated that researchers who present results back to Nunatsiavut communities get their results translated into Labrador Inuttitut; however, only about 10 percent of researchers come back to share their results with members of the communities. Most research applications included local partners; however, the inclusion of local partners on NGRAC applications had no bearing on whether data was returned.

## Discussion

Over the decade of Nunatsiavut-based research that we analyzed, there was a dramatic increase in research approved and conducted within Nunatsiavut as well as in researchers’ agreeing to return data to the Nunatsiavut Government. However, researchers’ data sovereignty commitments have not yet translated into increased Nunatsiavut data ownership. The lack of usable data and metadata included in returned data and results is concerning. Given the conversations we had with researchers in the process of this analysis, a lack of returned data or usable data was most often unintentional—a consequence of poor data management protocols. Secondarily, it was due to researchers prioritizing publishing results before returning data or results to community or to the Nunatsiavut Government. In the cases where not returning data was due to concerns over Nunatsiavut’s intention with said data, this mistrust is misplaced. Nunatsiavut Government’s stated goals for recovering data conducted within its communities and territory is to be able to better guide future researchers toward mutually beneficial projects, appropriately manage Nunatsiavut resources, and build community resilience (Nuesslein et al. 2021). While presenting this case study to settler or non-Indigenous researchers, they frequently asked about Nunatsiavut’s capacity to store and manage their data. This is a critical point that needs to be addressed. However, returning the data is not a question of capacity; it is a question of rights. Existing capacity inequities are the result of colonialist power structures that disproportionately benefit settler institutions. That ongoing injustice cannot and should not be the rationalization for denying data and research sovereignty, but rather the starting point to support ongoing community-based data management goals.

Given the preponderance of research relating to wildlife and resource management as well as climate, it is extremely important for the Nunatsiavut Government to have access to and ownership of this data to best develop adaptation and mitigation plans, build resiliency in the face of climate change, and manage its wildlife and natural resources. Since natural science research projects do not go through academic Research Ethics Board processes if they do not involve human participants, it is possible that research project leaders do not consider other ethics boards in their research development (Wong et al. 2020). This may explain why nine of the 10 research projects we identified that had no apparent connection to NGRAC approval process were in the natural sciences. However, animal ethics protocols and applications should be known to ecologists and biologists, and thus this may not fully explain the disconnect between natural science research and data return.

The call for Indigenous communities to own their own data is not new. It is well-established that access to data directly benefits communities and supports Indigenous sovereignty (Israel et al. 1998; Cheah et al. 2015; Chitondo and Dombroski 2019; Paul 2023). Instead of supporting Indigenous sovereignty, research has been, and continues to be, used as a tool to further colonialism (Hollowell and Julie 2007; Wilson 2008; ITK 2018). Within Inuit Nunangat, scientific research has reinforced Canadian state sovereignty and removed Inuit agency by relegating them to the objects of research or detached Inuit communities from the research taking place on their land and in their waters (ITK 2018). Current research practices still uphold these colonial legacies, and researchers not returning data and results to Nunatsiavut government and communities is one example of how this legacy persists.

On the other hand, researchers fulfilling their obligations to return data and results to Indigenous communities and governments advances Indigenous governance to the mutual benefit of communities and researchers (ITK and NRI 2006; Allemann and Dudeck 2019). To achieve these mutual benefits, we have compiled recommendations based on this case study to facilitate returning research data to Indigenous communities and further operationalize principles of Indigenous data ownership, control, access, and possession. Although these recommendations are drawn directly from this case study analysis, they are not new nor are they ours alone. Indigenous governments and communities have been instituting policies and publishing similar recommendations for decades (Carroll et al. 2020a). Our goal in outlining these recommendations is to respond directly to the issues we encountered within this case study. To do so, we delineate obligations and resultant recommendations to foster the research relationships among individual researchers—including students, institutions such as funding organizations, academia, and ethics boards, Indigenous research governance boards, and for all of us engaged in the process of data collection, use, manipulation, contextualization, and dissemination. While the recommendations we’ve compiled are mainly intended for non-Indigenous researchers doing work in communities other than their own, many researchers are Indigenous and working within their own communities and on their homelands. We intend for these recommendations to be useful for anyone committed to the principles of data sovereignty.

### Recommendations for Individual Researchers

**Learn the principles of data governance where you conduct your research**: Indigenous communities within the USA, Canada, Australia, and New Zealand have all published data sovereignty principles, guidance for conducting research respectfully, and standards regarding data ownership, control, access, and possession (Rainie and Rodriguez-Lonebear 2017; ITK 2018; Te Mana Raraunga 2022; The First Nations Principles of OCAP® 2023; Maiam Nayri Wingara 2024). It is every researcher’s individual responsibility to uphold and advance these principles when doing work with Indigenous communities or on Indigenous lands.

**Write research agreements that stipulate data management and ownership**: Formalizing researchers’ and community obligations to each other within a research project can and should include a conversation on how data will be treated. Doing so can build trust and collaboration between researchers and the communities in which they work (Woodward and Marrfurra McTaggart 2016; Government of Canada, Interagency Advisory Panel on Research Ethics 2023). Within this case study, researchers who informed us they had research agreements with Nunatsiavut returned their research at a higher frequency and often returned it before research publication.

**Include Indigenous ethics approval information in the methodology of your peer-reviewed journal articles and provenance and ownership of Indigenous data in the metadata**: When publishing research, make clear your obligations to community ethics boards (Jennings et al. 2023). Receiving Indigenous community or government approval of your research is part of research methodology and including that information in peer-reviewed journal articles helps to standardize this practice (Liboiron 2021, page 127). It also facilitates finding published results of research that has been conducted after receiving community approval.

**Write a research group data policy that includes plans for returning data and results**: Several researchers we spoke to did not know what happened to the data after they graduated if they were students, or after the students had graduated in the case of supervisors. Writing and maintaining a policy on data management is essential to ensure data are treated with care, maintain their secondary usefulness, and are returned to knowledge holders, as appropriate. Having data management contingency plans is especially important as students and researchers leave research labs and investigators retire. Data management and communication responsibilities with community continue when individuals move on, and plans should account for this. Individual researchers should also write their own data management protocol as part of their project’s methodology. This is especially important for research projects that do not undergo institutional ethics review to ensure researcher obligations to communities are well-defined regardless of the research topic.

**Include returning data and results in research funding proposals**: In conversations with researchers, many expressed concerns over finding funding to return to the communities to share results. It is imperative to properly fund the life of the research project to include returning data and results to communities (Tracy 2010). Funding should include means to transport and store data within the communities in which researchers work, as well as the means to return results to communities. Funding should support true knowledge mobilization within communities, not only within academic or policy circles (Roche et al. 2022). Therefore, funding should include methods and means to present research results to communities in a way that is culturally appropriate (Wong et al. 2020).

**Don’t wait until the study is published to return data and results**: Many NGRAC-approved researchers stated they could not return data or results until they were published. This policy is antithetical to good research. According to the National Inuit Strategy on Research, “Inuit representational organizations are the rightful gatekeepers of Inuit Nunangat research and are best positioned to determine how our information should be utilized and shared to maximize benefits and minimize harm” (ITK 2018). Sharing preliminary results with community members before publishing them also helps researchers to contextualize their results, an added benefit to their research (ITK and NRI 2006). In the case of the NGRAC process, researchers have an obligation to provide annual research summaries that outline where and how they collected data, and any relevant information that has come out of that data to date. In many cases, raw results would not be returnable until the conclusion of a research project. However, research summaries that include information on data collection are appropriate. Not returning any data or results until they are published makes it impossible for Indigenous communities and governments to help contextualize the data and provide useful feedback to researchers (Flowers 2023). It is inappropriate for communities to find out about studies that have been done in their communities from published journal articles (ITK and NRI 2006).

**Don’t call it “your” data**: Not returning data may be symptomatic of researchers’ deeper and perhaps unconscious assumption that they are the owners of the data with which they work. Researchers, and by extension the research relationships they build, would benefit from personal reflection interrogating the privilege of the assumption that researchers own Indigenous data. When researchers are working on Indigenous lands and with Indigenous knowledge, question your role as the data owner. Consider other terms that better capture researchers’ obligations in collecting, handling, and returning data to Indigenous communities and governance boards.

### Recommendations for Institutions

**Fund data management in Indigenous communities**: Fifty percent of Canada’s coastline and one-third of Canada’s landmass is within Inuit Nunangat (Simon 2011). Given the increase of research occurring within Inuit Nunangat, especially with the threat of climate change, there are immense data and storage needs to maintain sovereignty and accessibility of data (ITK 2018). Research funding bodies and academic institutions should be accounting for the financial responsibility inherent in good data management borne by sovereign entities within Inuit Nunangat when prioritizing research in the Arctic, and globally within Indigenous communities and territories.

**Hold researchers accountable for returning data and results**: Funding bodies, academic institutions, and government ministries all have the ability to ensure data and results are returned to communities. A plan to return data and research results can be part of funding and ethics applications. Additionally, funding bodies could make future grant awards contingent upon returning past data and results (Doering et al. 2022), similar to the way a report from a previously held grant is required before a researcher can apply to the same program in a new funding year.

**Local Indigenous research ethics should supersede other ethics requirements when research is conducted within Indigenous communities and in Indigenous territory**: The TCPS sets the standard for research involving Inuit, First Nations, and Métis within Canada, but it is not meant to supersede local Indigenous ethics (ITK 2018; Government of Canada, Interagency Advisory Panel on Research Ethics 2023). Despite this, some researchers who had conducted research with an approved NGRAC who stated they would return their data to Nunatsiavut told us they were unable to, due to perceived constraints placed on them by their research ethics board approval process at their academic institution. Although Canada’s TCPS standard has improved, international researchers do research with Inuit and within Inuit Nunangat and operate under differing ethics obligations to varying effects (Marley 2019). All potential impasses between institutional ethics boards and Indigenous ethics boards should be managed before research takes place with Indigenous peoples on Indigenous lands. In all cases, Indigenous ethical standards should be respected and followed.

**Include processes for Indigenous data access and data sharing in nation-state agencies, departments, and ministries**: Many research projects we evaluated were marine-based natural science research projects, and several of these involved researchers affiliated with Fisheries and Oceans Canada (DFO), the federal agency responsible for managing ocean resources. One of DFO’s long-term reconciliation strategy objectives is to recognize Indigenous self-determination by having “Indigenous groups effectively manage their own fisheries and other marine assets in their territories,” and “share in fisheries, oceans, aquatic habitat, and marine waterways decision-making” (Fisheries and Oceans Canada 2019). However, the strategy itself does not mention data and research sovereignty as means to this end. Data sovereignty plays an instrumental role in facilitating resource management and decision-making, and therefore should be included as means to achieving this long-term objective throughout governmental reconciliation strategies (James et al. 2014).

### Recommendations for Indigenous research governance boards

**Make returning data and results a condition of doing future research**: Researchers should always return their results to the communities in which they do research. To clarify guidelines over how good research is conducted, Indigenous communities can include returning results, and when appropriate, returning data, into the ethical requirements for doing research within their communities, on their lands, and in their waters.

**Set up structures for receiving returned data and keeping it safe and usable**: Some researchers we spoke to told us they had already returned their data in accordance with their data management policies, but we couldn’t locate it. With the increasing burden of managing and storing research data, it remains important to streamline the processes for receiving and safekeeping data to ensure they are not lost and are easy to retrieve and reuse, as appropriate.

**Require financial support for data management from multi-researcher, multi-institution research projects**: Large research projects with multi-institution research partnerships are becoming more common, especially in research partnerships investigating the effects of climate change (Jennings et al. 2023). In some cases, government-funded research projects have open-access data requirements or other data ownership stipulations that complicate Indigenous data sovereignty (Doering et al. 2022). Instead of being a barrier, these well-funded multi-year, multi-institution projects should support data sovereignty by financing Indigenous data management within the communities with which they work, especially given the additional burden these types of projects lay on Indigenous research institutions. Institutional financial support for Indigenous data sovereignty should not be conditional upon access to said data or be a tool to control data management processes; it should be a recognition for the additional workload of managing and coordinating these data-heavy projects.

## Conclusion

Regardless of which of the above overlapping categories one finds themselves, one must make returning data and results a priority when working in relationship with Indigenous Peoples, lands, and waters. To this end, it is important to consider the philosophical underpinnings of data ownership and shift toward a mindset of gathering and using data that precludes researcher ownership of data from Indigenous communities and homelands. In dominant scientific traditions, knowledge is extracted and decontextualized as data, which can then be owned and commoditized (McGregor 2021). In contrast, Indigenous knowledge, “is not just ‘knowledge’ (a noun) but a way of life, something that must be lived (a verb) in order to be understood” and is therefore “inseparable from the people who hold and live this knowledge” (McGregor 2021). Applying Indigenous epistemology precludes ownership of knowledge and should give researchers pause when considering their ownership of data collected in Indigenous communities, on Indigenous lands, and in Indigenous waters.

Ultimately, Indigenous peoples are best equipped to confront the adversities faced by their own communities, and are also the most effective stewards of their lands and waters (ITK 2018; IPBES 2019). Recognizing this, researchers have an obligation to facilitate self-governance rather than hinder it. A principal way of doing this is to ensure research data are owned and controlled by the Indigenous communities and organizations from where data originate. Furthermore, ongoing discussions and early data sharing between researchers and community can ensure that data are properly contextualized prior to publishing. Given the increase in research conducted on Indigenous lands and waters and with Indigenous communities, it is essential that effective institutional and individual strategies are put into place now. We hope that the case explored here can be helpful to other Indigenous organizations, communities, and tribal entities as they also navigate the complexities of research partnerships with settler institutions.

## Data Availability

All data needed to evaluate the conclusions in the paper are present in the paper. The Nunatsiavut Government Research Centre holds all raw data to comply with the principles of Indigenous data and research sovereignty. They are available upon request to future researchers. To request data, contact the Nunatsiavut Government Inuit Research Advisor.
